# Effects of enzymatic hydrolysis technology on the physicochemical properties and biological activities of American ginseng beverages

**DOI:** 10.1002/fsn3.4038

**Published:** 2024-03-18

**Authors:** Shengyuan Guo, Yichen Hu, Chaofan Zhao, Yajie Li, Zhuo Zhang, Wenting Wang, Yu Bai, Jiankang Zhou, Yajie Xue, Liang Zou, Guixing Ren

**Affiliations:** ^1^ College of Food and Bioengineering Chengdu University Chengdu China; ^2^ College of Life Science Shanxi University Taiyuan China

**Keywords:** American ginseng beverage, biological activities, enzymatic hydrolysis technology, physicochemical properties

## Abstract

American ginseng (*Panax quinquefolius L.*) contains various biological macromolecules, such as polysaccharides, saponins, and proteins, which have various pharmacological activities, including antioxidant, anti‐inflammatory, and hypoglycemic effects. Consequently, the utilization of novel processing technologies developed an American ginseng beverage to meet people's health needs and the preferences of young people. This study was the first to use American ginseng as a primary raw material, utilizing a three‐step enzymatic hydrolysis approach with cellulase, pectinase, amylase, maltase, and flavor protease enzymes to prepare an American ginseng beverage. The basic nutritional and active ingredient contents of the product were determined. The antioxidant activity of enzymatic beverages was evaluated by calculating the free radical clearance rates of DPPH and ABTS, and the effect of enzymatic beverages on α‐glucosidase activity was also tested. The anti‐inflammatory activity of RAW264.7 cells induced by LPS was evaluated by measuring the production of NO, TNF‐α, and IL‐6 during the enzymatic hydrolysis process. The results indicated that the nutritional components of American ginseng beverage products met the beverage industry standards. Moreover, the application of enzymatic hydrolysis technology had improved the antioxidant and anti‐inflammatory activities of American ginseng beverages. In addition, the enzymatic beverage of American ginseng exhibited certain hypoglycemic activity. Consequently, the established enzymatic hydrolysis technology provided a reference for the production of other beverage products.

## Graphical abstract

Due to the presence of various functional bioactive macromolecules in American ginseng, this study developed an American ginseng beverage with antioxidant, anti‐inflammatory, and hypoglycemic activities using a complex enzymatic hydrolysis technology. Additionally, the established enzymatic hydrolysis technology provided a reference for the production of other beverage products.

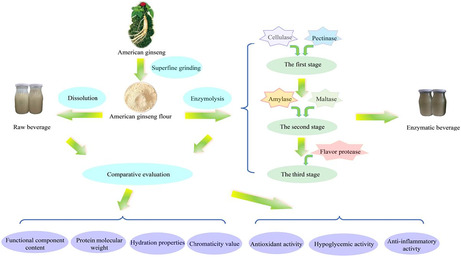

## 1 INTRODUCTION

*Panax quinquefolium*, a traditional Chinese medicinal herb, found its initial documentation in the Compendium of Materia Medica. Belonging to the Araliaceae family, this perennial herbaceous plant within the *Panax genus* possesses immense economic value, acclaimed for its “tonic” properties (Shuai et al., 2022). American ginseng thrives in suitable climate and soil conditions, typically situated between latitudes 30–47 degrees north. Originating from the primeval forests of North America, it is primarily cultivated in Ontario, Canada, and Wisconsin, USA (Nadeau & Olivier, 2003). Following its introduction for cultivation in the 1980s, numerous regions in China, including North China and Northeast China, have proven conducive to its growth requirements. Boasting a diverse range of chemical compositions, American ginseng presents a substantial abundance of saponins, polysaccharides, polyacetylenes, flavonoids, organic acids, and more. Consequently, it shows an extensive array of biological activities, including antioxidative, antifatigue, antitumor, anti‐inflammatory, and immune‐enhancing properties (Huang et al., 2022).

Through market research, it is found that the utilization of American ginseng spans across a wide spectrum, mainly by integrating its active ingredients into traditional Chinese medicine reagents, new drug formulations, and health drinks. Especially, the consumption of American ginseng in these domains exhibits a year‐on‐year increase, indicative of a promising outlook for American ginseng beverage products. Previous studies had delved into the realm of ginseng beverage production. For instance, Kim and colleagues introduced a composite health beverage by incorporating 2% red ginseng extract into an herbal medicine‐ginseng blend (Kim et al., 2019). Similarly, Li and colleagues focused on the creation of *Pueraria‐Ophiopogon* tea utilizing the primary constituents of American ginseng, *Pueraria*, and *Ophiopogon* (Li et al., 2023). Presently, prevailing American ginseng beverages in the market rely on extracts or leach liquor derived from American ginseng. However, this method suffers from a long extraction time, low raw material utilization, pronounced bitterness in the final product, and compromised overall flavor profile (Lopez‐Ochoa et al., 2022; Tárrega et al., 2012).

Presently, the application of new enzymatic hydrolysis technology has emerged as a promising approach in the realm of beverage production. This innovative technique enables the preservation of vital nutrients and functional compounds while concurrently enhancing the flavor profile of the final product. Especially, enzymatic hydrolysis has found successful application within the realm of cereal beverages (Tan et al., 2023). Utilizing the inherent specificity and selectivity of enzymatic reactions, biological enzymatic hydrolysis technology facilitates the selection of appropriate enzymes for hydrolysis or degradation based on the composition of plant cell walls. By destroying the structure of the cell wall, substances in the cells are dissolved, greatly increasing the content of active substances in the solvent (Pap et al., 2020). For instance, Rao and colleagues employed a combination of amylase and protease in the preparation of *Chrysanthemum*–*coix* seed beverage (Rao et al., 2023). Through detailed research, it was indicated that in comparison with conventional water extraction and ethanol extraction methods, the composite enzyme approach exhibited a significant improvement in the soluble sugar content, free amino acid content, and overall yield of instant Pu'er tea.

At present, the market lacks ginseng beverage products that have been prepared utilizing enzymatic hydrolysis technology. In this study, American ginseng was employed as the primary raw material, and a three‐step enzymatic hydrolysis process involving cellulase and pectinase, amylase and maltase, as well as flavor protease, was used to prepare American ginseng beverages. The physicochemical indicators of the enzymatic hydrolysis of American ginseng beverages were determined, while the biological activity of each stage of enzymatic hydrolysis in relation to American ginseng beverages was evaluated. Overall, the development of an American ginseng beverage with antioxidant, anti‐inflammatory, and hypoglycemic functions prepared using new enzymatic hydrolysis technology was aimed at meeting people's demand for healthy beverages, with the aim of promoting the development of the American ginseng industry and the wider application of enzymatic hydrolysis technology.

## 2 MATERIALS AND METHODS

### 2.1 Reagents

Dried American ginseng was purchased from Jinzi Agricultural Products Co., Ltd (Changchun, China). Cellulase (100,000 U/g) and Pectinase (100,000 U/g) were purchased from Dingyuan Food Technology Co., Ltd (Ningbo, China). α‐Amylase (480KNU/g), Maltase (6400MANU/g), and flavor protease (500LAPU/g) were purchased from Novozymes Investment Co., Ltd (Beijing, China). The enzymes used above were all obtained from microbial fermentation and were of food grade. Salt purchased from the market. Heavy calcium carbonate and tricalcium phosphate were purchased from Henan Wanbang Industrial Co., Ltd. 3,5‐Dinitrosalicylic acid (DNS) reagent was purchased from Kulaibo Technology Co., Ltd (Beijing, China). Vitamin C (VC) and α‐Glucosidase (100 U, CAS: 9001‐42‐7) were purchased from Beijing Solarbio Technology Co., Ltd (Beijing, China).

Ginsenoside standard was purchased from Jilin Ginseng Research Institute (Jilin, China). Protein prefabricated adhesive purchased from Lamboulide Trading Co., Ltd (Beijing, China). Lipopolysaccharide (LPS) was purchased from Sigma Aldrich, Mouse macrophage cell line, RAW264.7 and Penicillin Streptomycin were purchased from Shanghai Fu Heng Biotechnology Co., Ltd (Shanghai, China). Glucose test kit purchased from Zhongsheng Beikong Biotechnology Co., Ltd (Beijing, China). Fetal bovine serum (FBS) was purchased from Gibco Company in the United States (New York, USA). The other chemicals and reagents used in this study were of analytical grade.

### 2.2 Sample preparation

**FIGURE 1:**
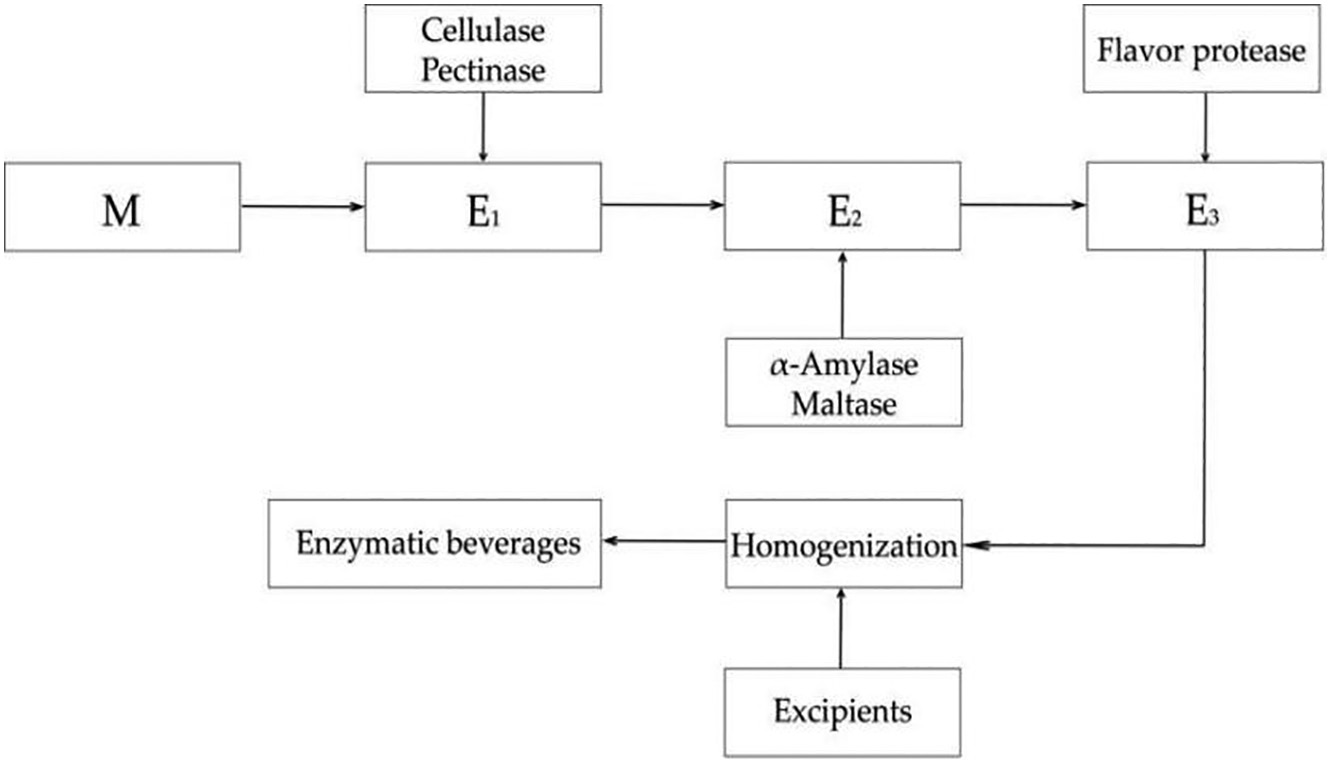
This is the American ginseng beverage process flow chart. E1: products obtained after hydrolysis of cellulase and pectinase; E2: products obtained after hydrolysis of α‐amylase and maltase; E3: products obtained after hydrolysis of flavor protease.

The raw material of American ginseng was used to obtain a finer powder by ultra‐micro‐grinder (Kangyuan Pharmaceutical Machinery Co., Ltd, Ruian, China) (six repetitions) and sieved through a 120 mesh sieve. After dissolution, the original solution of American ginseng was prepared, denoted as M. The American ginseng powder was enzymatically hydrolyzed in a ratio of 1:4 (g: mL, the same as below). The enzymatic hydrolysis process was divided into three steps: the first step was that 4.5% cellulase and pectinase were used to enzymatically digest cellulose and pectin to destroy the cell wall of the plant at 60°C for 2.25 h (de Souza & Kawaguti, 2021), and the material obtained after homogenization (High‐Pressure Homogenizer, Donghua Homogenizer Factory, Shanghai, China) was recorded as E1. The second step was that 5.5% α‐amylase and maltase were used to enzymatically digest starch at 65°C for 2.25 h (Tan et al., 2023), and the material obtained after homogenization was recorded as E2. The third step was that 1% flavor protease was used to remove part of the bitter peptide at 55°C for 1 h (Liu et al., 2022), and the material obtained after homogenization was recorded as E3. Following enzymatic hydrolysis, the material−liquid ratio was adjusted to 1:60, subsequently incorporating 0.5% rapeseed oil, 0.1% stabilizer (comprising calcium carbonate and tricalcium phosphate), and 0.1% salt (food grade), proportionate to the water volume. The mixture was homogenized (High‐Pressure Homogenizer, GYB40‐10SX, Donghua Homogenizer Factory, Shanghai, China) at a pressure of 30 MPa for 2 min, followed by an additional homogenization step at 60 MPa for 2 min to obtain the American ginseng enzymatic hydrolysis beverage final products. It was worth noting that the samples of E1, E2, and E3 referred to enzymatic hydrolysis products, rather than the final formulated beverages. The specific process flow is shown in Figure 1.

### 2.3 Basic nutrients

The basic nutritional components of American ginseng beverage products were determined employing established national standard methods, as shown in Table 1.

**TABLE 1:** National standard methods for determining basic nutritional components.

Project	Determination method
Energy	GB/Z 21922–2008
Protein	GB 5009.6–2016
Fat	GB 5009.6–2016
Dietary fiber	GB 5009.88–2014
Carbohydrate	GB/Z 21922–2008
Sodium	GB/Z 21922–2008
Moisture content	GB 5009.3–2016

### 2.4 Saponin content

The analysis of saponin content in the American ginseng beverage was executed utilizing a high‐performance liquid chromatography (HPLC) method. Each American ginseng beverage sample (1 g) was carefully weighed and individually combined with 20 mL of 70% methanol. Subsequently, an ultrasonic extraction was conducted at room temperature for 2 h. The resultant supernatant was then subjected to filtration using a 0.45‐μm filter membrane prior to injection. A fixed volume of 50 μL was injected in triplicate to ensure accuracy and precision of the analysis.

The chromatographic conditions were established based on our laboratory's validated methodology (Xue et al., 2017). A high‐performance liquid chromatograph (LC‐20AT) obtained from Shimadzu Company in Japan was utilized, equipped with an YMC‐ODS‐AM chromatographic column (4.6 mm × 250 mm, 5 μm). The mobile phases consisted of water (A) and acetonitrile (B), and the flow rate was set at 0.8 mL/min. Detection was performed at a wavelength of 202 nm, while the column temperature was maintained at 25°C. The elution gradient employed was as follows: initially, 25% B at 0 min, followed by 30% B at 15 min, further increased to 38% B at 24 min, held at 38% B until 28 min, then rose to 46% B at 30 min, subsequently rose to 74% B at 50 min, and finally, reached 100% B at 60 min.

### 2.5 Polysaccharide content

The Phenol sulfuric acid method was employed to determine the polysaccharide content of the American ginseng beverage. Anhydrous glucose was taken and dried to a constant weight in an oven (Yiheng Scientific Instrument Co., Ltd, Shanghai, China). Subsequently, 10 mg of the constant weight glucose was weighed and dissolved in a 100‐mL volumetric flask, followed by the preparation of a 0.1 mg/mL glucose standard solution using distilled water. Volumes of 0.2, 0.4, 0.6, 0.8, and 1.0 mL of the standard solution were then taken and supplemented with water to obtain a total volume of 1.0 mL. In these solutions, 0.5 mL of 6% phenol and 2.5 mL of concentrated sulfuric acid were added, mixed thoroughly, and left at room temperature for 20 min. The resulting absorbance was measured at 490 nm utilizing a multifunctional enzyme labeling instrument (Molecular Devices, USA). A standard curve was constructed by plotting the total polysaccharide content on the x‐axis and absorbance on the y‐axis. The sample was diluted to an appropriate extent, and subsequently, 1.0 mL was taken according to the calibration method.

### 2.6 Chromaticity value

Following a program developed in our laboratory, the colorimetric value of American ginseng beverage was measured using a spectrophotometer (NH‐110, Sanenchi Technology Co., Ltd, Shenzhen, China) (Qin et al., 2010). The brightness value (L*), red‐green value (a*), and yellow‐blue value (b*) were represented by the chromaticity value of the enzymatically hydrolyzed beverage sample. The total color difference (ΔE*) was calculated using the following Equation 1:

(1) $$∆E^{*}=\sqrt{{\left(∆L^{*}\right)}^{2}+{\left(∆a^{*}\right)}^{2}+{\left(∆b^{*}\right)}^{2}}$$

### 2.7 Hydration properties

The water absorption index (WAI) and water solubility index (WSI) of American ginseng beverage were determined following a previously established method with minor alterations (Zhang et al., 2023). Specifically, 2.00 g of the freeze‐dried (Vacuum freeze dryer, LGJ‐10, Beijing Songyuan Huaxing Biotechnology Co., Ltd) American ginseng beverage sample was taken and added to 25 mL of distilled water. The mixture was then soaked in water at 30°C for 30 min, being stirred every 10 min. The solution was then centrifuged at a speed of 5000 rpm (High‐speed centrifuge, H4‐20K, Hunan Kecheng Instrument Equipment Co., Ltd) for 15 min. The supernatant was subsequently dried to a constant weight in an oven. In Equations 2 and 3, *m*_0_ represents the mass of the sample, *m*_1_ indicates the mass of the supernatant after drying, and *m*_2_ denotes the sediment mass.

(2) $$\mathrm{WSI}=\frac{m_{1}}{m_{0}}\times 100\%$$

(3) $$\mathrm{WAI}=\frac{\left(m_{2}-m_{0}\right)}{m_{0}}$$

### 2.8 SDS‐PAGE analysis

The sample was diluted to the appropriate concentration using 12% resolving gel and 5% stacking gel with acrylamide content. A volume of 20 μL of the diluted sample was mixed thoroughly with an equal volume of buffer solution (50 μL β‐mercaptoethanol: 950 μL 2× Laemmli loading buffer). The resulting mixture was subjected to heat treatment in boiling water for 8 min, followed by a brief freezing period of 5 min. Subsequently, the prepared sample was loaded onto an electrophoresis analyzer (164–5070, BIO‐RAD Company, USA). Upon completion of the electrophoresis process, the gel was stained with Coomassie brilliant blue R‐250 for 30 min and subsequently destained with a transparent solution until the electrophoresis band became clearly visible.

### 2.9 Antioxidant activity

The determination of DPPH free radical scavenging rate followed a previously established protocol in our laboratory (Sharma et al., 2007). The samples M, E1, E2, and E3 were diluted into concentration gradients of 1 mg/mL, 2 mg/mL, 3 mg/mL, 4 mg/mL, and 5 mg/mL, respectively, and mixed thoroughly with equal volumes of DPPH. The resulting mixture was allowed to react for 30 min at room temperature, shielded from light. Subsequently, the absorbance was measured at 517 nm as *A*_1_ after the reaction, with a sample blank designated as *A*_2_, and a control group consisting solely of methanol and DPPH recorded as *A*_3_. VC was utilized as the most positive control in these experiments. The scavenging rate was determined using the Equation 4:

(4) $$R\%=\left(1-\frac{A_{1}-A_{2}}{A_{3}}\right)\times 100$$

The ABTS activity assay was conducted following a previously reported procedure with minor alterations (Yao et al., 2013). The samples M, E1, E2, and E3 were diluted into concentration gradients of 1 mg/mL, 2 mg/mL, 3 mg/mL, 4 mg/mL, and 5 mg/mL and thoroughly mixed with ABTS solution in a 1:4 ratio. After incubating for 5 min at room temperature, the absorbance was measured at 734 nm as *A*_1_. A sample blank was employed and its absorbance was recorded as *A*_2_. In the control group, 95% ethanol was utilized in place of the samples, and its absorbance was denoted as *A*_3_. The positive control for this experiment was Vc. The scavenging rate was determined using the Equation 5:

(5) $$R\%=\left(1-\frac{A_{1}-A_{2}}{A_{3}}\right)\times 100$$

### 2.10 α‐Glucosidase inhibitory activity

The samples were subjected to dilution across varying gradients, and 10 μL of each diluted sample was added to a 96‐well plate. Subsequently, 100 μL of a 2.5 U/mL glucosidase solution was added to initiate the reaction, which was carried out at 37°C for 10 min. Following this, an additional 36 μL of 0.06 M maltose was introduced to sustain the reaction for another 10 min. To terminate the reaction, 60 μL of 0.2 M sodium carbonate was added. Subsequently, 10 μL of the reaction mixture was taken and incubated with 1 mL of a glucose chromogenic agent at 37°C for 15 min. The absorbance was measured at 505 nm and recorded as *A*_1_. For the sample blank group, 0.1 M phosphate buffer solution was employed and denoted as *A*_2_. The control group replaced the sample with phosphate buffer solution, with one group containing added enzyme denoted as *A*_3_, and another group with only buffer labeled as *A*_4_. Acarbose was utilized as a positive control in this experiment. The scavenging rate was calculated using the Equation 6:

(6) $$\text{Inhibition}\;\left(\%\right)=\frac{\left(A_{4}-A_{3}\right)-\left(A_{1}-A_{2}\right)}{\left(A_{4}-A_{3}\right)}\times 100$$

### 2.11 Anti‐inflammatory activity

#### 2.11.1 Preparation of solution

A NaNO_2_ solution was prepared by dissolving NaNO_2_ in distilled water to create a 10 mM NaNO_2_ mother liquor. This mother liquor was further diluted to different concentrations (0 μM, 10 μM, 20 μM, 30 μM, 40 μM, and 50 μM) to generate the NO standard curve. Griess reagent was prepared by adding 250 mL of deionized water, ensuring thorough mixing, and allowing it to stand at room temperature for 30 min prior to utilization. Two milligrams per milliliter of LPS stock solution was prepared and subsequently diluted 100‐fold to obtain a final concentration of 20 μg/mL LPS reagent as required. The prepared LPS reagent was stored in a refrigerator at 4°C for future use. Ten percent of fetal bovine serum (FBS) and 1% double antibody Amphotericin B (Penicillin Streptomycin) were added to DMEM medium to prepare the complete medium. The resulting mixture was thoroughly mixed and subsequently stored in a refrigerator at 4°C for future use. A suitable quantity of raw material M and enzymatic hydrolysis products E1, E2, and E3 were taken and mixed with 70% ethanol at a ratio of 1:20, respectively. The mixture was subjected to ultrasonication for 2 h at room temperature, followed by centrifugation and concentration before freeze‐drying. Upon completion of the lyophilization process, the samples were kept in a refrigerator at 4°C for future use. Prior to use, the freeze‐dried sample was dissolved in culture medium and further diluted to concentrations of 1 mg/mL, 2 mg/mL, 3 mg/mL, 4 mg/mL, and 5 mg/mL as the test samples.

#### 2.11.2 Cell culture and treatment

Mouse monocyte–macrophage line RAW264.7 cells were cultured in a CO_2_ incubator using a prepared DMEM complete medium. Once the cells reached confluence in the culture flask, they were passaged as follows: Firstly, the original medium was discarded, and the cells were rinsed twice with PBS to eliminate any floating cells. Subsequently, an appropriate volume of fresh medium was added, and the adherent cells were gently scraped off using a scraper. The cells were then resuspended by pipetting with a pipette gun to ensure uniform suspension, after which they were placed back into culture for continued growth.

#### 2.11.3 Cytotoxicity assay

Cell viability was assessed using the standardized CCK‐8 method. RAW264.7 cells in the logarithmic growth phase were washed twice with PBS, resuspended in the culture medium, and gently detached from the culture surface using a spatula. The cell suspension was homogenized by pipetting with a pipette gun to ensure uniform distribution and a specific volume of the cell suspension was obtained for cell counting. Upon adjusting the cell density, 100 μL of cells was seeded into each well of a 96‐well cell culture plate. The plate was then placed in a CO_2_ incubator under conditions of 5% CO_2_, 37°C, and saturated humidity for 24 h. Subsequently, 10 μL of samples with varying concentrations was added to respective wells, and after 6 h of further incubation, 10 μL of CCK‐8 reagent was introduced. The plate was returned to the CO_2_ incubator for 1 h. Absorbance at 450 nm was measured and recorded as *A*_1_. A blank control group without samples was used to record the absorbance as *A*_2_, while the cell culture medium served as the sample blank group and was recorded as *A*_3_. Cell survival rate was calculated using the Equation 7:

(7) $$\text{Cell viability}\;\left(\%\right)=\frac{A_{1}-A_{3}}{A_{2}-A_{3}}\times 100$$

#### 2.11.4 NO measurement

The determination of American ginseng beverage samples was determined as follows: RAW264.7 cells during logarithmic growth were rinsed twice with PBS and suspended in medium with an appropriate volume. The walled cells were then gently detached using a spatula, and cell density was adjusted to 2.5 × 106 cells/mL through pipetting and the addition of medium. Subsequently, 100 μL of the cell suspension and 100 μL of different concentrations of American ginseng beverage samples were added to each well of a 96‐well cell culture plate. The plate was placed in a CO_2_ incubator for 2 h. Following this, the LPS solution was introduced to the sample group and the control group to reach a final concentration of 1 μg/mL. The blank group was treated similarly but with an equivalent volume of medium. After 22 h of further incubation, 50 μL of supernatant from each group was transferred to a new 96‐well cell culture plate, followed by the addition of 50 μL of Griess reagent. The reaction was allowed to proceed at 37°C for 15 min, after which absorbance was measured at 540 nm on an enzyme marker. The NO production in the supernatant was calculated accordingly. The levels of TNF‐α and IL‐6 were determined according to the ELISA assay kit instructions.

### 2.12 Statistical analysis

The experiment was conducted in triplicate and the results are presented as Mean ± SD (SD, standard deviation). Statistical analysis was performed using SPSS version 26.0 (SPSS Inc., Chicago, IL, USA). The data were subjected to one‐way analysis of variance (ANOVA) to determine the differences between samples. Significant differences were compared by Duncan test on the level of *p* < .05.

## 3 RESULTS AND DISCUSSION

### 3.1 Basic nutrient content

The nutritional content of American ginseng beverages is shown in Table 2. According to the national standard for solid drinks, the moisture content should not exceed 7%. In this study, the moisture content of the American ginseng beverage was determined to be 5.84%, satisfying the national standard requirements. Notably, ginseng protein possesses various benefits including promoting cell proliferation, exerting antitumor effects, delaying aging through antioxidant mechanisms, enhancing immune function, and lowering blood lipid levels (Gong et al., 2020; Lam & Ng, 2002). The protein content in the beverage was measured at 9.96%, whereas the raw American ginseng material contained 13.44% protein. Although a fraction of the protein may be lost during enzymatic hydrolysis, a relatively significant portion still remains, enabling its biological activity. Moreover, dietary fiber plays a crucial role in increasing satiety, preventing diabetes, reducing the risk of cardiovascular diseases, and regulating gut microbiota (Anudeep et al., 2016; Jovanovski et al., 2021). The American ginseng beverage exhibited a comparatively high level of dietary fiber, measuring 21.55 g/100 g and constituting 86% of the Nutrient Reference Value (NRV).

**TABLE 2:** Basic nutrient content of American ginseng beverage.

Parameter	Every 100 g (g)	Nutrient reference value (NRV) %
Energy	1444 ± 0.21 (kJ)	17%
Protein	9.96 ± 0.05 (g)	17%
Fat	15.60 ± 0.12 (g)	26%
Dietary fiber	21.55 ± 0.11 (g)	86%
Carbohydrate	30.8 ± 0.02 (g)	10%
Sodium	191 ± 0.06 (mg)	9.5%
Moisture content	5.84 ± 0.07 (g)	ND

*Note*: The unit g/g represents the proportion of nutrients in the final enzymatic beverage product.

### 3.2 Saponin content

At present, over 620 types of ginsenosides have been isolated from Panax plants, with more than 40 varieties identified specifically in American ginseng (Piao et al., 2020). Among these, five major ginsenosides were primarily detected in American ginseng beverage, namely, Rg1, Re, Rb1, Rc, and Rd. However, Rg1 and Re were not individually separated but combined as Rg1 + Re. As shown in Table 3, the total saponin content in M was 2.58%, and the total saponin content in E1, E2, and E3 was 1.34%, 1.78%, and 1.48%, respectively. However, the total saponin content of the products obtained from the three enzymatic hydrolysis processes had all decreased. Saponins consist of saponin ligands and sugars, such as uronic acid or other organic acids. Enzymatic hydrolysis may cause the cleavage of glycosyl groups within saponins, leading to a decrease in their overall content, similar to what has been described in LI (Li & Fan, 2020). Notably, treatment with α‐amylase and maltase resulted in an increase in E2 saponin content compared to E1. Conversely, the use of flavor protease led to a decrease in E3 saponin content, still higher than that of E1. This result may be attributed to flavor protease primarily acting upon proteins and bitter peptides, potentially causing the decomposition of saponins over time. Importantly, the change trend for individual monomer saponin content was consistent with the total saponin content.

**TABLE 3:** Content of saponin monomers in American ginseng beverage.

Saponin category	Standard	*R* ^2^	M (mg/g)	E1 (mg/g)	E2 (mg/g)	E3 (mg/g)
Rg1 + Re	Y = 94,441x + 82,297	.9993	8.82a ± 0.21	4.49c ± 0.03	5.84b ± 0.01	4.91c ± 0.03
Rb1	Y = 117,294x + 42,053	.9992	10.6a ± 0.18	4.77d ± 0.11	6.29b ± 0.04	5.31c ± 0.12
Rc	Y = 39,587x + 36,331	.9993	3.00a ± 0.13	1.51 ± 0.07c	2.09 ± 0.04b	1.84 ± 0.07c
Rd	Y = 57,907x + 73,047	.9997	3.35 ± 0.04a	2.60 ± 0.02b	3.54 ± 0.05a	2.78 ± 0.02b
Total saponins (Rg1 + Re + Rb1 + Rc + Rd)			25.77 ± 0.40a	13.38 ± 0.22d	17.76 ± 0.03b	14.84 ± 0.16c

*Note*: Data are presented as mean ± standard deviation. Different letters (a, b, c, d) indicate significant differences (*p* < .05). The unit mg/g represents the proportion of saponins in the enzymatically hydrolyzed products (E1, E2, and E3) content.

### 3.3 Polysaccharide content

**FIGURE 2:**
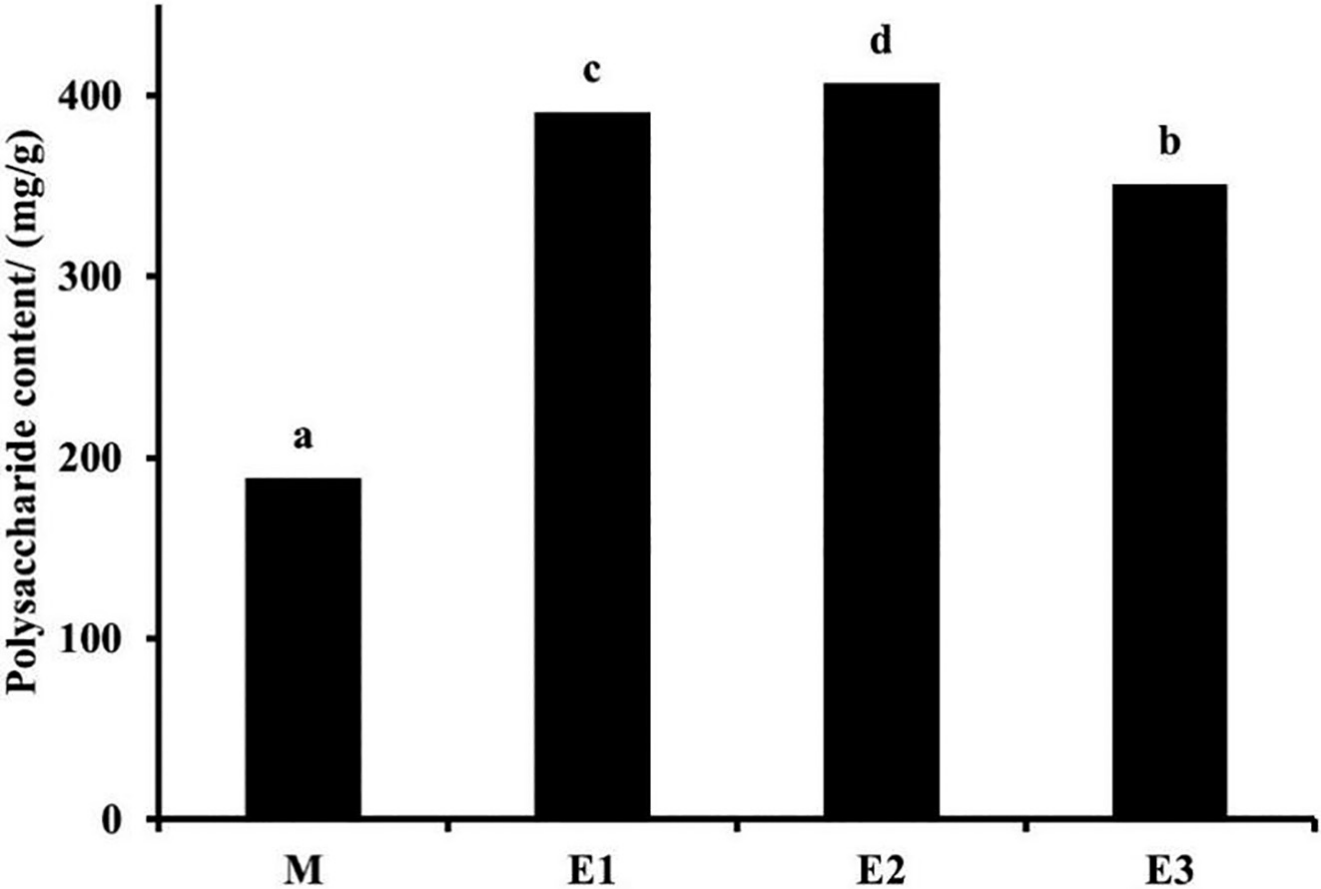
Polysaccharide content of different American ginseng beverage samples. Data are presented as mean ± standard deviation. Different letters (a, b, c, d) indicate significant differences (*p* < .05). The unit mg/g represents the proportion of polysaccharides in the enzymatically hydrolyzed products (E1, E2, and E3) content.

American ginseng polysaccharides, as one of the main activities of American ginseng, exhibit many effects including immune enhancement, antitumor activity, and glycemic control (Ren et al., 2023). As shown in Figure 2, enzymatic hydrolysis had been observed to enhance the polysaccharide content within American ginseng beverages. The reason may be due to the degradation of cell walls and membranes by enzymes, which reduced the resistance generated by the solvent extraction process, promoting the release of polysaccharides. Concurrently, enzymes further degraded certain polysaccharides into smaller molecular weight fragments, thereby favoring the separation of polysaccharides from the cell (Nadar et al., 2018). The initial two steps of enzymatic hydrolysis displayed an upward trend in polysaccharide content, while the subsequent third step demonstrated a reduction in polysaccharide levels. Following the completion of the enzymatic reaction, the polysaccharide content exhibited no increase with time. When the enzyme was sufficient, an extended duration of enzymatic hydrolysis led to the degradation of polysaccharides into monosaccharides by the enzyme, resulting in a reduction in their overall content (Chen et al., 2014). Notably, the trends observed of polysaccharide content during enzymatic hydrolysis mirrored those observed for saponins.

### 3.4 Chromaticity value

Color is a crucial quality parameter that exerts a direct influence on consumer acceptance. These color attributes are indicative of starch caramelization and the Maillard reaction, which transpires between sugars and amino acids (Özer, 2022). As illustrated in Table 4, the L* value exhibited a gradual descent, while both the a* and b* values displayed a progressive ascent. These trends implied a darkening brightness of American ginseng beverages, coupled with an intensification of redness along the red‐green axis and a deepening of yellowness along the yellow–blue axis. This result could be attributed to the caramelization and Maillard reaction transpiring within the sample during high‐temperature enzymatic hydrolysis, thereby augmenting the coloration of the final product. Comparable findings had been reported in earlier investigations, elucidating the degradation of pigments and nonenzymatic browning reactions as explanatory factors (Lund & Ray, 2017). Additionally, certain studies have emphasized that a ΔE* value exceeding 1 denotes perceptible color alterations discernible to the naked eye (Lee et al., 2019). Based on the table, the calculated ΔE* value unequivocally signified visible color changes instigated by enzymatic hydrolysis in comparison with the raw material of American ginseng.

**TABLE 4:** Chromaticity value of different American ginseng beverage samples.

Sample	L* (bright‐dark)	a* (red‐green)	b* (yellow‐blue)	c* (chrominance)	h* (hue angle)	ΔE*
M	83.82 ± 0.16a	1.89 ± 0.08c	15.61 ± 0.24c	15.72 ± 0.25c	83.11 ± 0.19a	–
E1	76.50 ± 0.62b	3.16 ± 0.03b	22.23 ± 0.05b	22.45 ± 0.06b	81.91 ± 0.06b	49.68 ± 5.30c
E2	72.98 ± 1.13c	3.29 ± 0.28b	23.10 ± 0.45b	23.33 ± 0.49b	81.89 ± 0.54b	88.25 ± 6.03b
E3	66.83 ± 0.94d	4.95 ± 0.08a	25.49 ± 0.17a	25.97 ± 0.18a	79.01 ± 0.10c	198.11 ± 9.54a

*Note*: Data are presented as mean ± standard deviation. Different letters (a, b, c, d) indicate significant differences (*p* < .05).

### 3.5 Hydration properties

**FIGURE 3:**
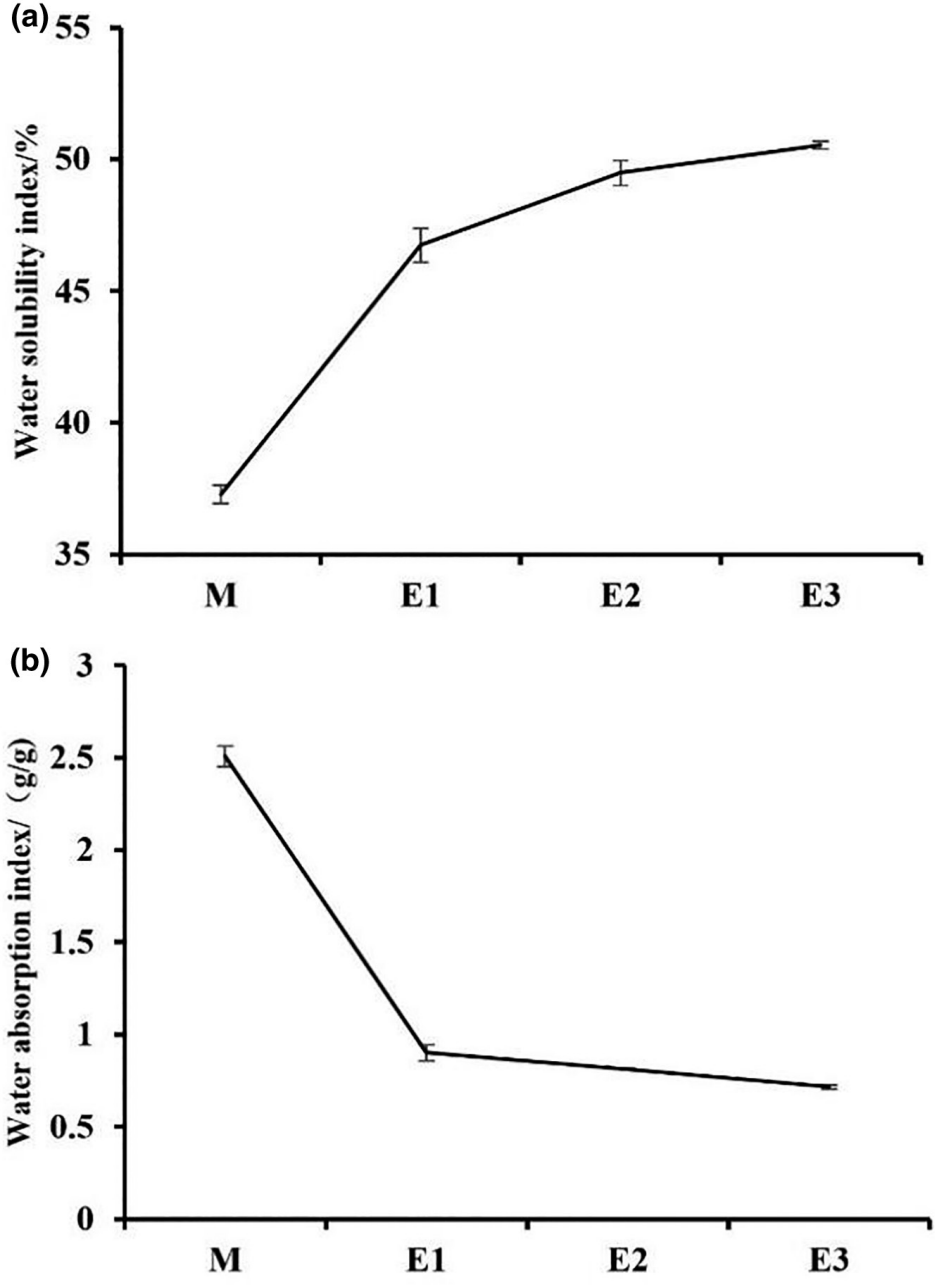
WSI of different American ginseng beverage samples: (a). WAI of different American ginseng beverage samples: (b). Data are presented as mean ± standard deviation. WAI, water absorption index. WSI, water solubility index.

The WAI and WSI scores are widely acknowledged as reliable indices for assessing the extent of starch gelatinization and degradation (Sun et al., 2019). As depicted in Figure 3, notable differences were observed in the WSI and WAI values of American ginseng beverages following enzymatic hydrolysis treatment. Specifically, enzymatic hydrolysis engendered an upsurge in the water solubility index, as evidenced by the WSI value. The increase in enzymatically treated samples was more pronounced compared to their raw counterparts, while the rise in samples treated with successive enzymatic hydrolysis was slower. This rise in WSI value can be attributed to the breakdown of starch and protein into smaller molecules during enzymatic hydrolysis, thus rendering it more soluble. In terms of the WAI value, enzymatic hydrolysis treatment led to a reduction in the water absorption index. Significantly, samples subjected to enzymatic hydrolysis displayed a marked decline in WAI value compared to untreated American ginseng beverages, whereas samples treated with successive enzymatic hydrolysis exhibited a more gradual fall. The WAI value was indicative of the integrity of starch in aqueous dispersion, which hinges on the availability of hydrophilic groups that can combine with water molecules along with the gel‐forming ability of amylose (Zhang et al., 2023). The reduction in WAI value may be attributed to the destruction of hydrophilic groups and the gel‐forming capacity of amylose consequential to amylase hydrolysis of American ginseng beverages.

### 3.6 SDS‐PAGE results

**FIGURE 4:**
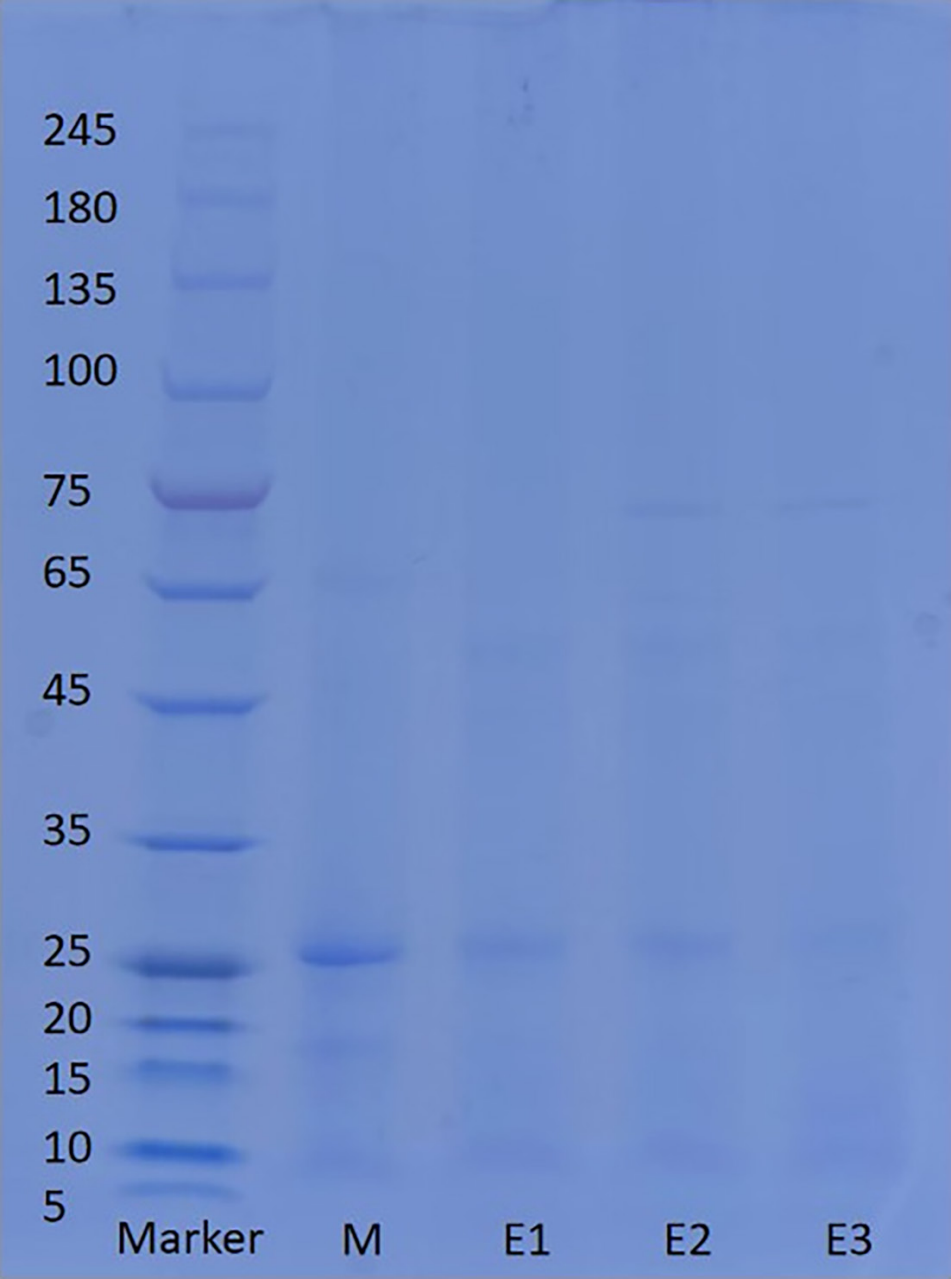
Electrophoretic map of different American ginseng beverage samples.

From the electrophoresis map (Figure 4), it was evident that the protein bands observed in the sample were primarily distributed within the range of 5–75 kDa. On the other hand, the protein bands present in the raw materials of American ginseng could be detected at 10 kDa, 15–20 kDa, and 65 kDa. Notably, the protein bands at 25–35 kDa exhibited enhanced clarity. Previous investigations had revealed distinct bands at 24 kDa, 21 kDa, 20 kDa, 16 kDa, and 12 kDa, thereby forming a characteristic fingerprint specific to American ginseng (Sun & Chen, 2011). Following the initial stage of enzymatic hydrolysis, the protein bands at 10–20 kDa and 65 kDa vanished, while the bands at 10 kDa and 25 kDa exhibited reduced intensity. Additionally, new bands emerged within the range of 45–65 kDa. The possible reason for the degradation of protein bands at 25 kDa was due to the decomposition of cellulose into glucose, and the decomposition of pectin into monosaccharides such as rhamnose, galacturonic acid, arabinose, and galactose. These monosaccharides formed glycosylation products with proteins, thereby affecting the depolymerization of protein molecules (Teng et al., 2021). Subsequently, during the second phase of enzymatic hydrolysis, a novel band appeared at 75 kDa, distinguishing it from the previous stage, whereas no substantial differences were observed in comparison with the remaining bands. In contrast to preceding steps, the third stage of enzymatic hydrolysis displayed shallower bands without any new or disappeared bands. Presumably, the absence of certain protein bands could be attributed to the enzymatic hydrolysis process, whereby large molecule proteins were broken down into smaller peptide molecules. The newly formed protein bands were likely associated with the introduced enzymes. The differential alterations in protein bands subsequent to enzymatic hydrolysis were likely attributable to the distinct actions of various enzymes.

### 3.7 Antioxidant activity

**FIGURE 5:**
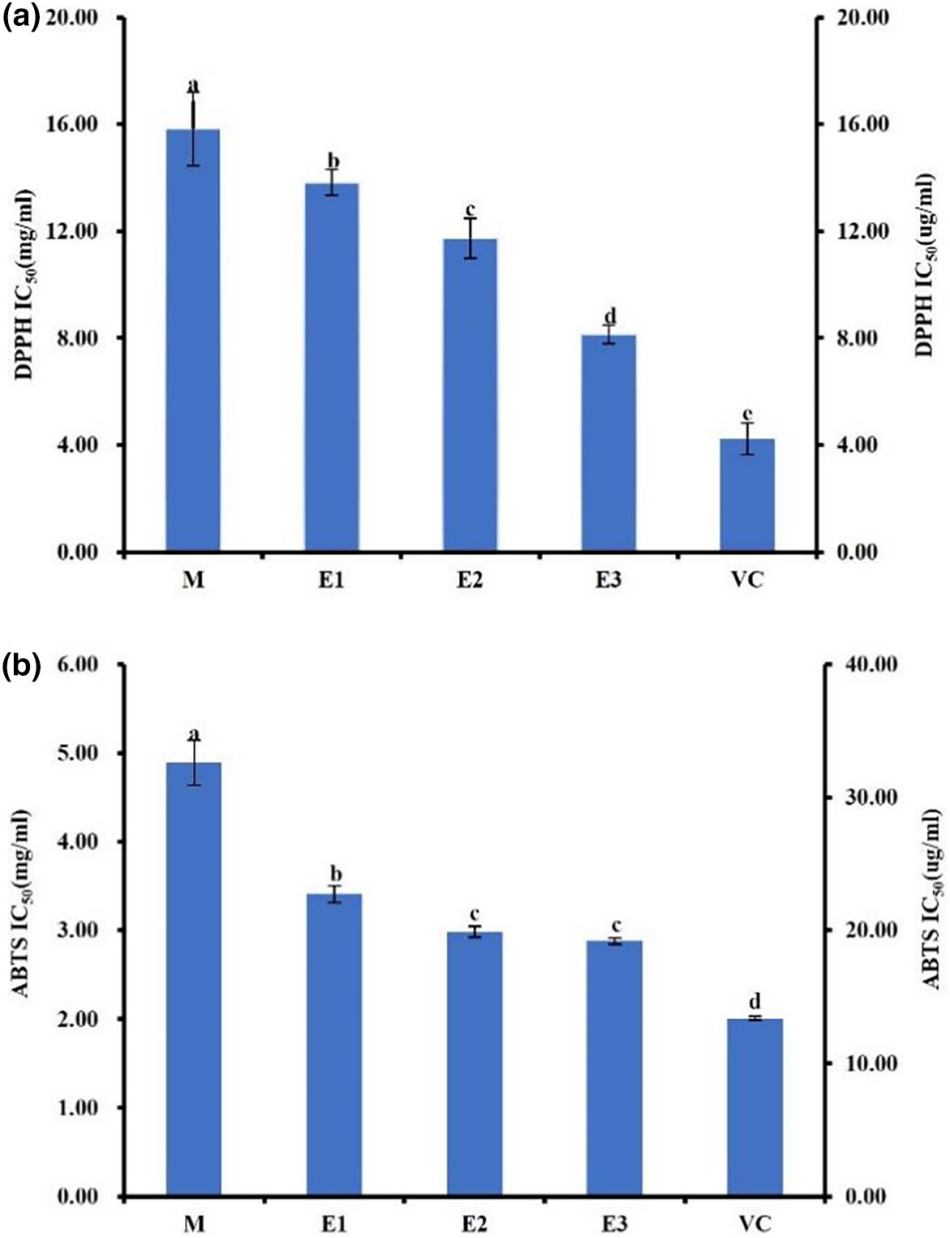
The antioxidant activities of different American ginseng beverage samples and reference substance: (a) DPPH radical scavenging activity. (b) ABTS radical scavenging activity. The left y‐axis represents the IC50 scale of the sample, and the right y‐axis represents the IC50 scale of the VC. Data are presented as mean ± standard deviation. Different letters (a, b, c, d, e) indicate significant differences (*p* < .05).

During the metabolic process, the human body invariably generates free radicals, and an excess of these radicals can induce substantial damage to the organism (Harman, 2002). Counteracting the adverse effects of excessive free radicals, antioxidants have been demonstrated to exert pronounced efficacy. Two different measurement approaches, namely, DPPH and ABTS, were adopted in this study to assess the antioxidant activity of American ginseng beverages subjected to distinct enzymatic hydrolysis processes. As depicted in Figure 5, the IC50 values of samples M, E1, E2, and E3 were determined to be 15.83 mg/mL, 13.82 mg/mL, 11.73 mg/mL, and 8.14 mg/mL, respectively. The IC50 value of the positive control, VC, was measured to be 4.23 ug/mL. Notably, the enzymatic hydrolysis treatments significantly heightened the antioxidant activity of the American ginseng beverage, with a concomitant increase observed in such activity as the enzymatic hydrolysis stage progressed. The enhanced antioxidant activity of enzymatic hydrolysis products E1 and E2 was likely related to the increase in polysaccharide content in the products after enzymatic hydrolysis, which was consistent with the trend of changes in polysaccharide content in section 3.3 of the article. Ginseng polysaccharides have an antioxidative ability and can scavenge superoxide free radicals and hydroxyl free radicals (Luo & Fang, 2008). Ginseng neutral polysaccharides have stronger antioxidant activity than acidic polysaccharides (Chen & Huang, 2019), but further exploration is needed for the types of polysaccharides in enzymatic beverages. Jia and colleagues found that an acid polysaccharide (WGPA‐A) can increase the levels of catalase and superoxide dismutase (SOD) in order to scavenge hydroxyl free radicals. Additionally, this polysaccharide protected the mitochondrial integrity of the red gastrocnemius muscle (Wang et al., 2014).

The enhanced antioxidant activity of enzymatic hydrolysis product E3 may be related to many antioxidant peptides formed by enzymatic hydrolysis. The enzymatic hydrolysis procedure represented a favored strategy for preparing antioxidant peptides (Ashaolu, 2020), as evidenced by Lv's research, which indicated that macromolecule‐hydrolyzed antioxidant peptides from food sources could confer biological activities on the resultant peptides (Lv et al., 2022). These findings were consistent with those obtained in our current investigation. In this investigation, American ginseng beverages were subjected to treatment with a range of enzymes, leading to the generation of small‐molecule substances exhibiting enhanced antioxidant activity. In a similar vein, Shanmugam et al. (2015) examined the hydrolysis of buffalo casein using pepsin, trypsin, and chymotrypsin individually and in combination and observed that peptides derived from pepsin–trypsin hydrolysis displayed the highest antioxidant activity. This observation underscored the enzyme‐specific characteristics that contributed to the production of peptides with heightened activity, thereby emphasizing the potential benefits associated with employing multiple enzymatic hydrolysis approaches.

In the foreseeable future, the trend of employing a combination of suitable enzymes for more efficient enzymatic hydrolysis is anticipated. As illustrated in Figure 5, the ABTS free radical assay yielded outcomes comparable to those obtained using DPPH. The IC50 values determined for samples M, E1, E2, and E3 during ABTS analysis were 4.89 mg/mL, 3.41 mg/mL, 2.98 mg/mL, and 2.87 mg/mL, respectively. The IC50 value of the positive control, Vc, was recorded as 13.38 ug/mL. Notably, the antioxidant capacity of American ginseng beverages evaluated via the ABTS method exhibited a significant elevation compared to that assessed using the DPPH method, thus aligning with prior investigations. Furthermore, enzymatic hydrolysis facilitated the conversion of macromolecular constituents present in American ginseng drinks into small‐molecule substances, such as glucose, amino acids, and peptides. Subsequently, these small‐molecule substances underwent nonenzymatic catalysis, yielding a series of Maillard reaction products characterized by enhanced antioxidant activity (Fu et al., 2020).

### 3.8 α‐Glucosidase inhibitory activity

**FIGURE 6:**
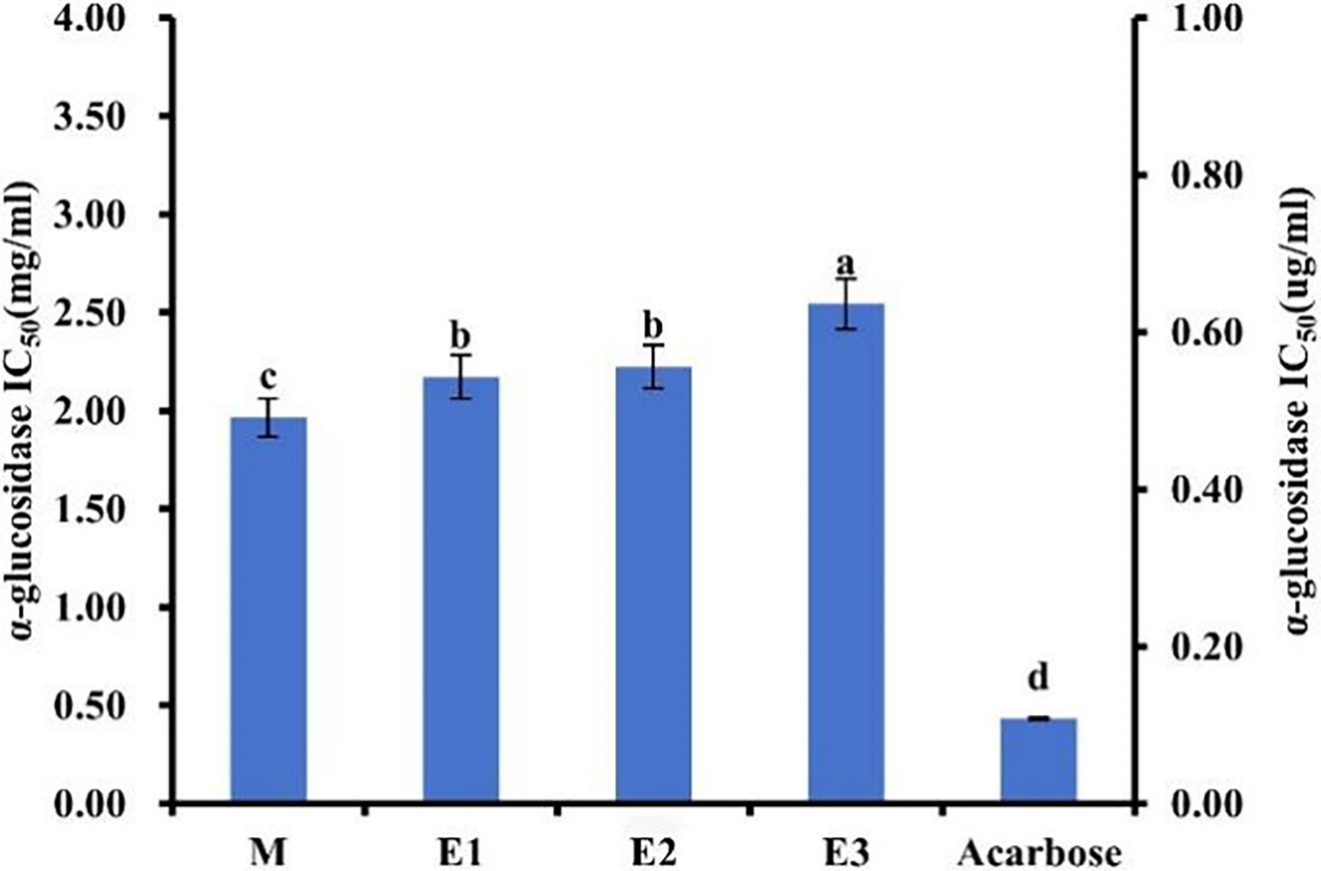
The α‐glucosidase inhibitory activities of different American ginseng beverage samples. The left y‐axis represents the IC50 scale of the sample, and the right y‐axis represents the IC50 scale of the acarbose. Data are presented as mean ± standard deviation. Different letters (a, b, c, d) indicate significant differences (*p* < .05).

α‐Glucosidase assumes a crucial role in the hydrolysis of dietary carbohydrates. By competitively binding to enzyme binding sites on small intestinal brush border epithelial cells, this enzyme inhibitor effectively delays the production of monosaccharides, ultimately leading to stabilized postprandial blood glucose levels (Xu et al., 2018). To investigate the α‐glucosidase inhibitory activity of American ginseng beverages following various enzymatic hydrolysis processes, Figure 6 displayed the experimental findings. Upon meticulous calculating, the IC50 values for samples M, E1, E2, and E3 were determined as 1.96 mg/mL, 2.17 mg/mL, 2.22 mg/mL, and 2.54 mg/mL, respectively. Additionally, Acarbose served as the positive control in this study, exhibiting an IC50 value of 108.27 ng/mL. Notably, no significant variance in the inhibitory effect on α‐glucosidase activity was observed among the different enzymatic hydrolysis treatments applied to American ginseng beverages. This may be due to the considerable increase in glucose content resulting from enzymatic hydrolysis, surpassing the reduction in glucose caused by the inhibitory action of American ginseng beverages on α‐glucosidase, falsely showing no inhibitory effect on α‐glucosidase by American ginseng beverages. The α‐glucosidase activity of the enzymatic hydrolysis products in the third stage slightly increased, possibly due to a decrease in the content of saponins and polysaccharides with hypoglycemic effects. In addition, the IC50 value of the final enzymatic product E3 was only 2.54 mg/mL, which also indicated that the American ginseng enzymatic beverage had α‐glucosidase inhibitory activity.

In addition, it was credible that saponins and polysaccharides derived from American ginseng represented the primary active constituents responsible for the inhibition of α‐glucosidase activity. Studies conducted in vitro and in vivo had indicated that ginsenosides possessed the potential to enhance energy expenditure through activation of the adenosine monophosphate‐activated kinase pathway, while also exhibiting the ability to reduce energy intake in a comparable manner (Ratan et al., 2021). In a study conducted by Wang and colleagues, it was demonstrated that a ginseng polysaccharide led to significant reductions in blood glucose and glucagon levels (Wang et al., 2019).

### 3.9 Anti‐inflammatory activity

#### 3.9.1 Cytotoxicity

**FIGURE 7:**
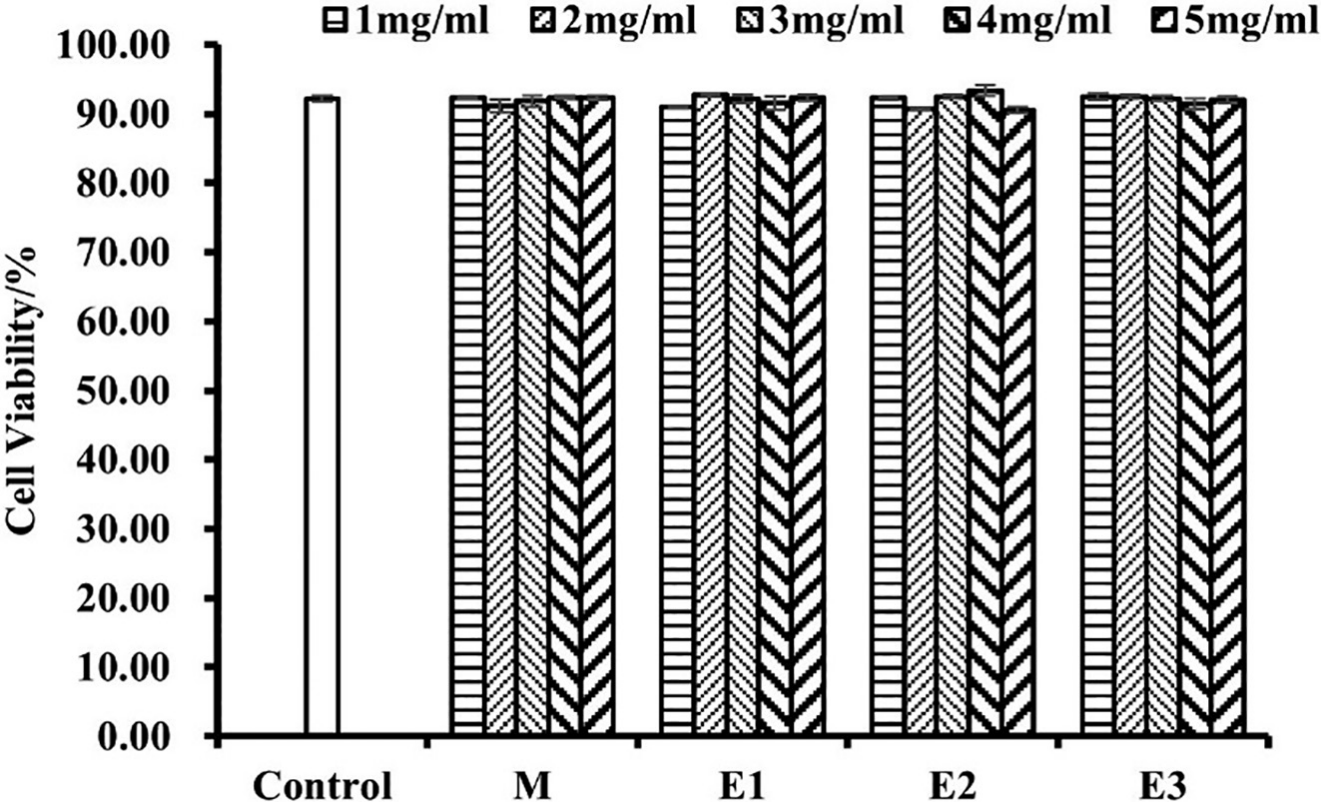
Cytotoxic effect of American ginseng beverage samples on mouse RAW264.7 macrophage cells. Data are presented as mean ± standard deviation. The unit mg/mL represents the concentration of enzymatically hydrolyzed American ginseng powder in the enzymatically hydrolyzed beverage products (E1, E2, and E3).

The effects of different enzymatic treatments on the growth of RAW264.7 cells as detected by CCK‐8 cell proliferation and toxicity assay kit are shown in Figure 7. From the figure, it can be seen that the different enzymatic treatments of American ginseng beverage in the series of concentration gradients (5 mg/mL, 4 mg/mL, 3 mg/mL, 2 mg/mL, and 1 mg/mL) had no significant effects on the viability of mouse RAW264.7 cells, and none of the samples had significant effects on the viability of mouse RAW264.7 cells with increasing sample concentrations. Therefore, it indicated that the western ginseng beverage had no significant cytotoxicity, and the interference of sample toxicity on inflammation inhibition was excluded.

#### 3.9.2 NO release

**FIGURE 8:**
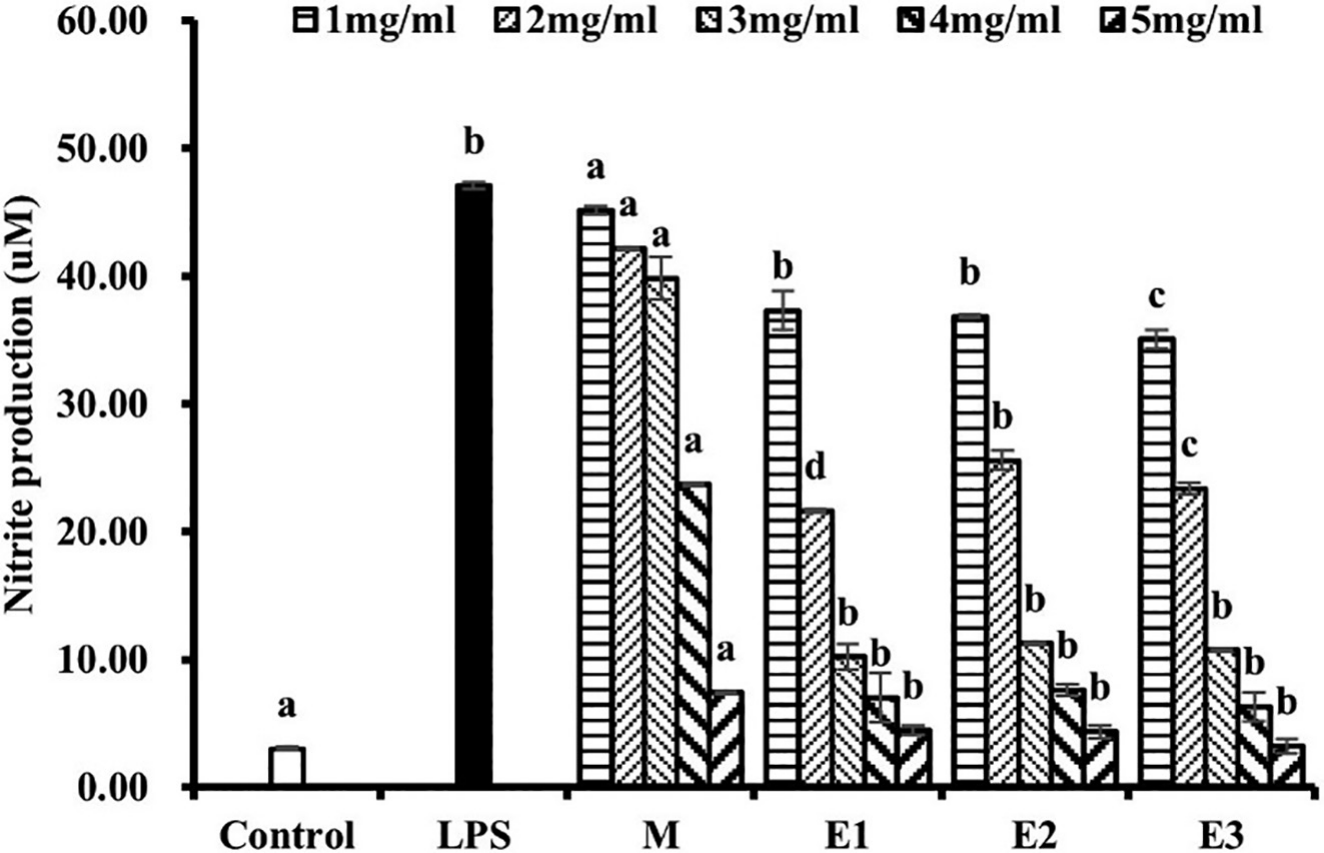
Inhibition effects of American ginseng beverage samples on NO production by LPS‐stimulated RAW 264.7 cells. Data are presented as mean ± standard deviation. Data are presented as mean ± standard deviation. Different letters (a, b, c, d) indicate significant differences (*p* < .05).

When macrophages are stimulated by lipopolysaccharide (LPS), an upregulation of NO and related cytokines is induced, leading to an inflammatory response and subsequent cytopathic tissue damage. Thus, modulating the overproduction of NO and its related cytokines triggered by LPS becomes crucial in anti‐inflammatory therapy (Sharma et al., 2007). The quantification of macrophage immunity and inflammatory response primarily relies on the assessment of NO release, a critical indicator of macrophage activation. This study aimed to investigate the anti‐inflammatory potential of American ginseng beverage by evaluating its impact on LPS‐induced NO release from RAW264.7 cells. As depicted in Figure 8, LPS exhibited the capacity to enhance NO release and incite an inflammatory reaction within the cells, thereby serving as the positive control group. The release of NO from RAW264.7 cells in the blank group was 2.98 μm, and the LPS group was 47.08 μm. However, the American ginseng beverage, across a series of concentration gradients (5 mg/mL, 4 mg/mL, 3 mg/mL, 2 mg/mL, and 1 mg/mL), significantly suppressed NO production in a dose‐dependent manner when compared to the LPS group. Especially, the figure indicated that enzymatic treatment enhanced the anti‐inflammatory activity of the American ginseng beverage, although without significant variation among the different enzymatic treatments regarding their impact on anti‐inflammatory efficacy.

The enhanced anti‐inflammatory activity of enzymatic beverages was likely mainly related to the increase in polysaccharide content during the enzymatic hydrolysis process. Previous studies indicated that ginseng polysaccharides possessed anti‐inflammatory properties (Guo et al., 2021). Wang and colleagues used HT‐29 and HCT‐116 cell lines as models to study a ginseng berry polysaccharide. This polysaccharide was found to reduce the secretion of interleukin‐8. It also inhibited the production of Th1 and regulatory T cells to reduce inflammation (Wang et al., 2020). In addition, although the saponin content decreased during the enzymatic hydrolysis stage, various ginsenosides had anti‐inflammatory properties and could also affect the anti‐inflammatory activity of beverages (Im, 2020; Kang et al., 2018; Phi et al., 2019). Moreover, nonsaponin components extracted from ginseng had been reported to repress the expression of inflammatory cytokines (Baek et al., 2016), while ginseng‐derived ingredients displayed a broad spectrum of anti‐inflammatory activities (Cho et al., 2023).

#### 3.9.3 TNF‐α and IL‐6 release

**FIGURE 9:**
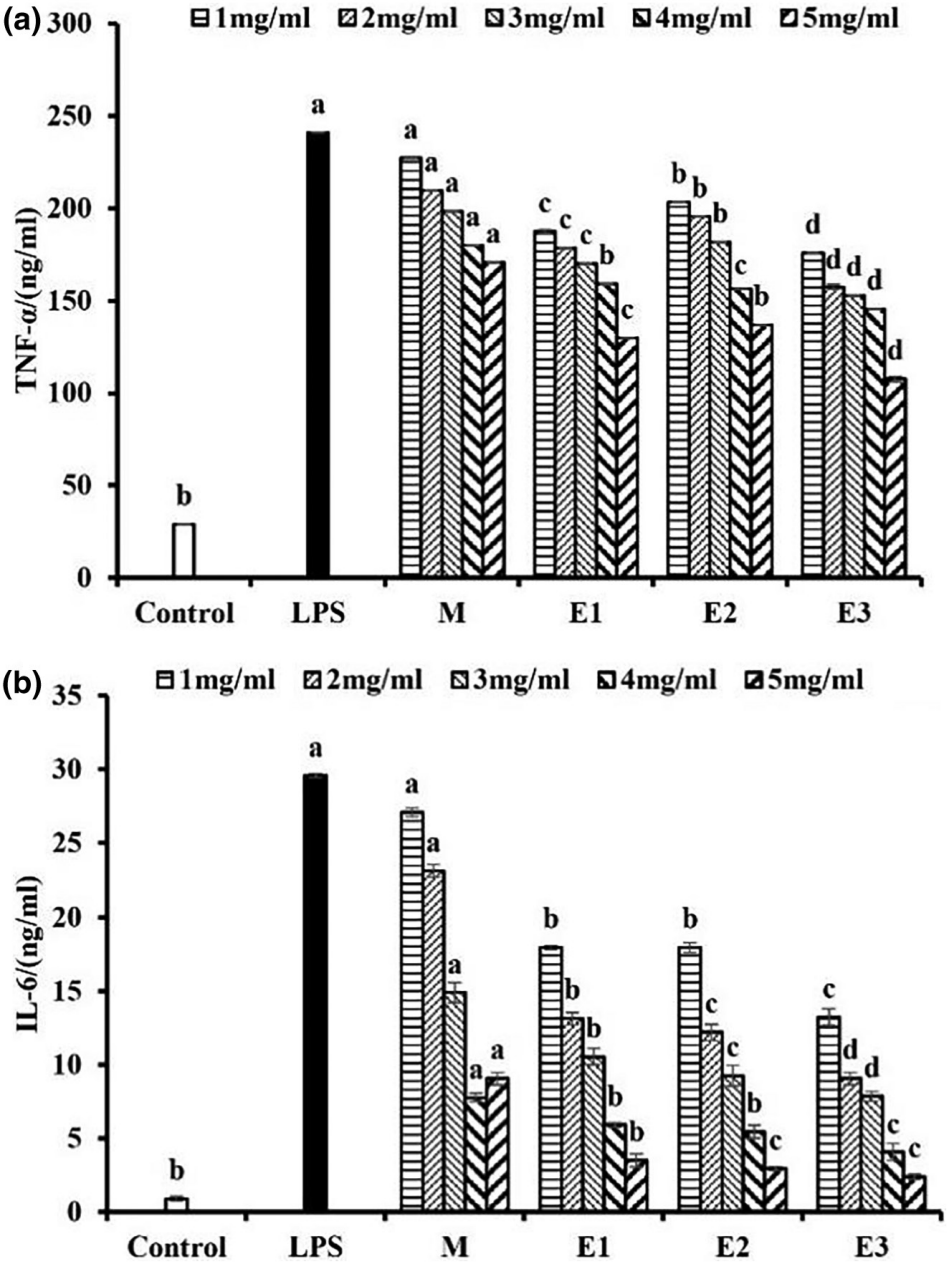
Effect of American ginseng beverage samples on LPS‐induced proinflammatory cytokine production: (a) TNF‐α and (b) IL‐6 expressions. Data are presented as mean ± standard deviation. Different letters (a, b, c, d) indicate significant differences (*p* < .05).

Cytokines are pivotal regulators of inflammation, and their involvement in chronic inflammation has been well established. IL‐6 has been observed to be upregulated in chronic inflammatory diseases, and TNF‐α is known to play a fundamental role in the pathological processes of inflammatory diseases (Turner et al., 2014). Therefore, the downregulation of these cytokines is considered a key function of anti‐inflammatory agents. The findings regarding the impact of American ginseng beverage on LPS‐induced tumor necrosis factor TNF‐α and IL‐6 in RAW264.7 cells are presented in Figure 9. It was evident that LPS led to an increase in TNF‐α and IL‐6 release, eliciting an inflammatory response within the cells. Hence, the LPS‐treated group was employed as a positive control. In comparison with this group, the American ginseng beverage demonstrated a notable dose‐dependent inhibition of TNF‐α and IL‐6 production across various concentration gradients (5 mg/mL, 4 mg/mL, 3 mg/mL, 2 mg/mL, and 1 mg/mL). The American ginseng beverage generated through the utilization of enzymatic hydrolysis technology demonstrated a remarkable augmentation in its anti‐inflammatory activity, but the effect of different enzymatic treatments was not significant. The inhibitory effect on TNF‐α and IL‐6 was likely related to the functional active components in the enzymatic hydrolysates of American ginseng, such as saponins, polysaccharides, etc. In this regard, Ji and colleagues demonstrated that 70% ethanol extracts of hydroponic ginseng and soil‐cultivated ginseng reduced mRNA expression of TNF‐α, IL‐6, and IL‐1β (Hwang et al., 2018). Similarly, Jin and colleagues showed that the aqueous extract of Korean red ginseng had the capacity to inhibit the expression of IL‐1β, IL‐8, TNF‐α, and other mRNAs in the kidney, lung, liver, stomach, and colon of adult mice (Kim et al., 2021).

The NF‐κB signaling pathway has been extensively associated with various inflammatory mediators, including NO, TNF‐α, and IL‐1β (Lawrence, 2009). Moreover, apart from the NF‐κB signaling pathway, the mitogen‐activated protein kinase (MAPK) signaling pathway has also been recognized for its involvement in the generation of diverse inflammatory mediators. Therefore, our subsequent research will focus on elucidating the anti‐inflammatory pathway of the American ginseng beverage, aiming to gain deeper insights into its anti‐inflammatory mechanisms. Additionally, further comprehensive investigations pertaining to its clinical applications are warranted.

## 4 CONCLUSIONS

This study presented the pioneering inquiry into the enzymatic hydrolysis‐based preparation of American ginseng beverage. Enzymatic hydrolysis technology preserved important nutrients and functional compounds in American ginseng, decomposing cellulose, pectin, and starch in the raw materials to produce a sweet taste, enhancing the flavor of the final product, and making the consumer younger. Enzymatic hydrolysis‐based preparation of American ginseng beverage had been demonstrated to exert a significant enhancement in both antioxidant and anti‐inflammatory capacities. Notably, the American ginseng beverage also displayed hypoglycemic activity, but further proof of the strength of the activity was needed. Therefore, the beverages can be used as functional foods to prevent diseases related to inflammation and oxidative stress, thereby promoting the development of the American ginseng industry. Moreover, the established enzymatic hydrolysis technology exhibits promising prospects for the manufacturing of other beverage products in the industrial domain.

## AUTHOR CONTRIBUTIONS

**Shengyuan Guo:** Conceptualization (equal); methodology (equal); writing – original draft (equal). **Yichen Hu:** Conceptualization (equal); methodology (equal); writing – original draft (equal). **Chaofan Zhao:** Investigation (equal); methodology (equal). **Yajie Li:** Data curation (equal); methodology (equal). **Zhuo Zhang:** Conceptualization (equal); supervision (equal). **Wenting Wang:** Investigation (equal); project administration (equal). **Yu Bai:** Conceptualization (equal); visualization (equal). **Jiankang Zhou:** Investigation (equal); software (equal). **Yajie Xue:** Investigation (equal); visualization (equal). **Liang Zou:** Project administration (equal); supervision (equal); validation (equal). **Guixing Ren:** Funding acquisition (equal); project administration (equal); resources (equal); validation (equal).

## CONFLICT OF INTEREST STATEMENT

The authors declare no conflict of interest.

## Data Availability

All data generated or analyzed during this study are included in this manuscript.
